# Resveratrol suppresses OSCC invasion and migration by regulating macrophage polarization via Syk signaling pathway

**DOI:** 10.3389/fimmu.2025.1660851

**Published:** 2025-09-29

**Authors:** Weibo Li, Ying Qi, Yafei Li, Lu Liu, Xiaodan Dong, Bo Li

**Affiliations:** Department of Oral Anatomy and Physiology, Jilin Provincial Key Laboratory of Oral Biomedical Engineering, Hospital of Stomatology, Jilin University, Changchun, China

**Keywords:** oral squamous cell carcinoma, tumor-associated macrophages, resveratrol, polarization, invasion, migration

## Abstract

**Introduction:**

An increasing amount of evidence indicates that the metastasis in oral squamous cell carcinoma (OSCC) is closely associated with the polarization phenotype of tumor-associated macrophages (TAMs). Resveratrol (RES) has been demonstrated to exert an inhibitory effect on the invasion and migration of OSCC cells. However, the mechanism by which RES inhibits OSCC invasion and migration remains to be fully elucidated.

**Methods:**

RES for reprogramming TAMs (R-RES group) and RES group were used to interfere with the polarization of tumor-associated macrophages (TAMs). RT-qPCR, ELISA, Western blotting, immunofluorescence staining, transwell and wound-healing assays were used to investigate the anti-tumor mechanism of RES.

**Results:**

R-RES reprogramed TAMs from M2 to M1 phenotype. RES promoted M1 polarization of TAMs and inhibited M2 polarization of TAMs. In mechanism, inhibition of Syk signaling pathway in TAMs attenuated the invasive and migratory ability of CAL27 cells through promoting M1 polarization of TAMs and inhibiting M2 polarization of TAMs.

**Conclusions:**

RES suppresses OSCC invasion and migration by regulating the polarization phenotype of TAMs via Syk signaling pathway, further elucidating the anti-tumor mechanism of RES.

## Graphical abstract

RES suppresses OSCC invasion and migration by regulating the polarization phenotype of TAMs via Syk signaling pathway. RES reprograms TAMs from M2 to M1 phenotype. Meanwhile, it promotes M1 polarization of TAMs and inhibits M2 polarization of TAMs.

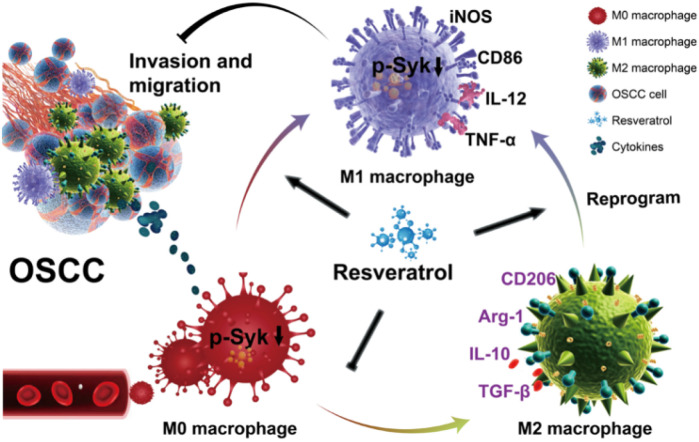

## 1 Introduction

Oral squamous cell carcinoma (OSCC) constitutes the sixth most prevalent malignant tumor globally, exhibiting a proclivity for cervical lymph node metastasis (1, 2). The five-year survival rate of OSCC is below 50%, which can be attributed to a lack of comprehensive understanding of its invasion and migration mechanisms (3, 4). It is urgent to further elucidate the mechanism of OSCC invasion and migration, identify new specific targets, and optimize treatment strategies to reduce OSCC mortality. An increasing body of evidence indicates that OSCC invasion and migration are closely associated with tumor-associated macrophages (TAMs) (5–7).

TAMs within the tumor microenvironment can be categorized into two main phenotypes: M1 (anti-tumor) and M2 (pro-tumor) (8–10). M2-type TAMs play a critical role in facilitating OSCC invasion and migration (11, 12) and are closely correlated with the survival rate of OSCC patients (13). Previous studies reported that M2-type TAMs can be reprogrammed into M1-type TAMs, effectively inhibiting tumor invasion and migration (14–17). Given the plasticity of the TAM phenotype, the repolarization of immunosuppressive and pro-tumor TAMs into immunostimulating and anti-tumor TAMs through pharmacological stimulation represents a promising strategy for tumor immunotherapy (18–20). In addition, promoting M1 polarization of TAMs while blocking M2 polarization of TAMs is also a valuable therapeutic strategy (10, 17, 21).

Plant extracts exhibit anti-tumor activity through different mechanisms (22–24). Resveratrol (RES) is a non-toxic, natural polyphenolic compound extracted from plants such as grapes, peanuts, and *Polygonum cuspidatum*, exhibiting an anti-tumor effects by regulating oxidative stress and glucose metabolism (21, 25, 26). RES has been demonstrated to exert an inhibitory effect on OSCC invasion and migration via p53/SLC7A11 (27). RES was found to suppress the invasion and migration of cisplatin-resistant OSCC cells by downregulating the expression of phosphorylated ERK/p38 and MMP-2/9 (28). RES restrained *Fusobacterium nucleatum*-induced EMT and migration by reducing SNAI1 expression (29). However, the regulatory role of RES on TAMs and its mechanism in the tumor microenvironment (TME) have not been fully elucidated. Zhang et al. revealed that RES effectively suppressed hepatocellular carcinoma progression by inhibiting TAM/M2 macrophage polarization and activation of the STAT3 pathway, while increasing IFN-γ-expressing effector CD8+ T cells in tumor-bearing mice (30). Chen et al. demonstrated that low-dose resveratrol is effective in controlling renal cell carcinoma growth through (i) inhibition of immunosuppressive cells, (ii) induction of activated and cytotoxic CD8+ T cells, and (iii) modulation of cytokine balance and angiogenesis in the tumor microenvironment (31). Cheuk et al. demonstrated that treatment with RES reduced the impact of TAM-derived IL-6 on breast cancer progression by promoting M1 polarization of macrophages (32). In the LLC mice treated with RES, the expression of M2 TAM markers (e.g., IL-10, Arg1, and CD206) was significantly reduced (33). RES may intervene in cancer progression by modulating the polarization state of TAMs and interrupting the interaction between tumor cells and macrophages (34).

Spleen tyrosine kinase (Syk) is a non-receptor tyrosine kinase. Emerging studies have recently shown that Syk also contributes to tumor progression (35). Rohila et al. demonstrated that genetic deletion or pharmacological inhibition of Syk using R788 induces a pro-inflammatory state in macrophages (36). In a pancreatic ductal adenocarcinoma *in vivo* model, the M2-like TAM phenotype was significantly reduced by a Syk inhibitor (37). Nevertheless, the role of the Syk pathway in TAM polarization remains unclear.

In this study, RES interfered with the polarization of TAMs in two ways. RES for reprogramming TAMs (R-RES) was added after 24 h induction of macrophages with the conditioned medium of CAL27 cells (CAL27-CM). RES and CAL27-CM were added simultaneously to observe the change in the polarization phenotype of TAMs. The purpose of this study was to investigate whether RES could suppress OSCC invasion and migration by regulating the polarization phenotype of TAMs, and to reveal its specific molecular mechanism.

## 2 Materials and methods

### 2.1 Cell culture

The human tongue squamous carcinoma cell line CAL27 and the mouse peritoneal macrophage cell line RAW264.7 were purchased from the China Center for Type Culture Collection (CCTCC) and subcultured at the Oral Experimental Teaching Center of Jilin University. The cells were cultured in DMEM (Gibco, USA) supplemented with 10% fetal bovine serum (FBS; BI, Israel) and 1% penicillin-streptomycin double antibody (BI, Israel) in a humidified atmosphere of 5% CO_2_ at 37°C.

### 2.2 Collection and preparation of CAL27-CM and induction of TAMs

CAL27 cells were uniformly grown to 80% confluence, washed, and the medium replaced. After 24 h, the supernatant was aspirated, centrifuged at room temperature, filtered through a sterilized 0.22-μM filter, and stored at −20°C. At the time of use, the tumor supernatant was pre-warmed and mixed with fresh medium at a 7:3 ratio to prepare CAL27-CM for subsequent experiments.

### 2.3 Cell viability assay

In 96-well plates, RAW264.7 cells and TAMs (5 × 10^5^ cells/ml) were incubated with RES (0 μM, 2.5 μM, 5 μM, 10 μM, 20 μM, and 40 μM) (Sigma, Germany) for 24 h. CCK-8 solution (10 μl) was added to each well, and the cells were incubated at 37 °C for 4 h. The optical density (OD) at 450 nm was measured using a microplate reader (BioTek, Vermont, USA).

### 2.4 Real-time quantitative PCR

Total RNA was extracted from cells using an RNA Extraction Kit (Takara, Japan) and reverse-transcribed into cDNA according to the manufacturer’s instructions. For RT-qPCR amplification of TNF-α, IL-12, iNOS, IL-10, Arg-1, and TGF-β from TAMs, targeted sequences were amplified using DNA polymerase. The relative expression of TAM cytokine mRNA was calculated using the 2^−ΔΔCT^ method. **Table 1** lists the primers used in this study.

**Table 1:** Primers used for RT-qPCR.

Gene	Forward primer 5′-3′	Reverse primer 5′-3′
TNF-α	CTCATGCACCACCACCAAGGACTC	AGACAGAGGCAACCCGACCACTC
TGF-β	GCAACAATTCCTGGCGTTACCTTG	CAGCCACTGCCGTACAACTCC
IL-12	CCTGTGACACGCCTGAAGAAGATG	CTTGTGGAGCAGCAGATGTGAGTG
IL-10	CTGCTATGCTGCCTGCTCTTACTG	ATGTGGCTCTGGCCGACTGG
iNOS	TGCCACGGACGAGACGGATAG	CTCTTCAAGCACCTCCAGGAACG
Arg-1	TGCTCACACTGACATCAACACTCC	GGTCTACGTCTCGCAAGCCAATG
β-actin	GTGCTATGTTGCTCTAGACTTCG	ATGCCACAGGATTCCATACC

### 2.5 Enzyme-linked immunosorbent assay

Groups were set according to different experimental purposes (control, CAL27-CM, R-RES, GS-9937, and RES). RES interfered with TAM polarization in two ways. R-RES refers to RES used for reprogramming TAMs. RAW264.7 cells were seeded in six-well plates, and the interventions were as follows. Control: normal complete medium was added. CAL27-CM: CAL27-CM was added to induce for 24 h. R-RES: cells were induced with CAL27-CM for 24 h and then replaced with 20 μM RES. GS-9937: 1 μM GS-9937 was added, and cells were induced with GS-9937-containing CAL27-CM for 24 h, then replaced with fresh medium. RES: cells were induced with CAL-CM containing 20 μM RES for 24 h, after which the medium was replaced with fresh medium. After 24 h, supernatants were collected to measure IL-10 and TNF-α levels using an enzyme immunoassay kit (CUSABIO, China) according to the manufacturer’s instructions.

### 2.6 Immunofluorescence staining

After fixation with 4% PFA, TAMs were blocked with 5% BSA (Solarbio, China). CD86 and CD206 antibodies (Thermo Fisher, Massachusetts, USA) were added and incubated overnight at 4°C. A red fluorescent secondary antibody (Thermo Fisher, Massachusetts, USA) was applied incubated in the dark for 1 h. Cells were washed with PBS and stained with DAPI (Thermo Fisher, Massachusetts, USA) for 2 min in the dark. Images were captured using an IX-71 fluorescence microscope (Olympus, Japan).

### 2.7 Transwell assay

RAW264.7 cells (1 × 10^5^/well) were seeded into 24-well 8.0 μm pore size inserts (Corning, USA). After different treatments, CAL27 cells (2 × 10^4^) were seeded into 24-well BioCoat Matrigel invasion chambers (Corning, USA), with Matrigel pre-coated in advance for the invasion test. After 24 h (migration) or 48 h (invasion), CAL27 cells were fixed, stained with crystal violet, and photographed under a microscope. Experimental results were quantified and analyzed using Image J.

### 2.8 Wound-healing assay

A wound healing culture insert (IBIDI, Germany) was placed into a six-well plate with 1 × 10^4^ CAL27 cells per insert. After cell attachment, the insert was removed. After washing with PBS, TAMs-CM was added and incubated for 24 h. Scratch closure at 0 h and 24 h was recorded using a microscope (Olympus, Japan).

### 2.9 Survival analysis

The OncoLnc tool (www.oncolnc.org) was used to perform overall survival analysis for patients with head and neck squamous cell carcinoma (HNSCC). The 50th (upper) and 50th (lower) percentiles were considered as Syk-high and Syk-low groups. All HNSCC data were obtained from TCGA. Using these data, the association between Syk expression and survival time in patients with HNSCC was analyzed.

### 2.10 Western blotting

Total proteins from TAMs after RES intervention were extracted with RIPA lysis buffer (P0013B) containing PMSF (ST505) and phosphatase inhibitor cocktail A (P1081) (all from Beyotime, China). Proteins were separated by 10% sodium dodecyl sulfate–polyacrylamide gel electrophoresis and transferred to a PVDF membrane (Bio-Rad, California, USA). After blocking with 5% nonfat milk, membrane-bound proteins were probed overnight at 4°C with primary antibodies (1:1,000) against Syk (CST, Massachusetts, USA), p-Syk (CST, Massachusetts, USA), and β-actin (CST, Massachusetts, USA), followed by incubation for 1 h at room temperature with secondary antibodies (1:2,000; CST, Massachusetts, USA). Antibody-bound protein bands were detected using enhanced chemiluminescence reagents (Bio-Rad, California, USA) and visualized with an automatic chemiluminescence/fluorescence imaging system (Tanon, China). Data were analyzed using Image J.

### 2.11 Statistical analysis

Data analysis was performed using GraphPad Prism 8. Each experiment was repeated three times. Data are presented as mean ± SD (n = 3). For normally distributed data, t-test was used to compare two groups, and a one-way ANOVA was used to compare multiple groups. A *P*-value <0.05 after correction was considered statistically significant. *P <0.05; **P <0.01; ***P <0.001; **** *P <*0.0001.

## 3 Results

### 3.1 CAL27-CM induced M2 polarization of TAMs

**Figure 1:**
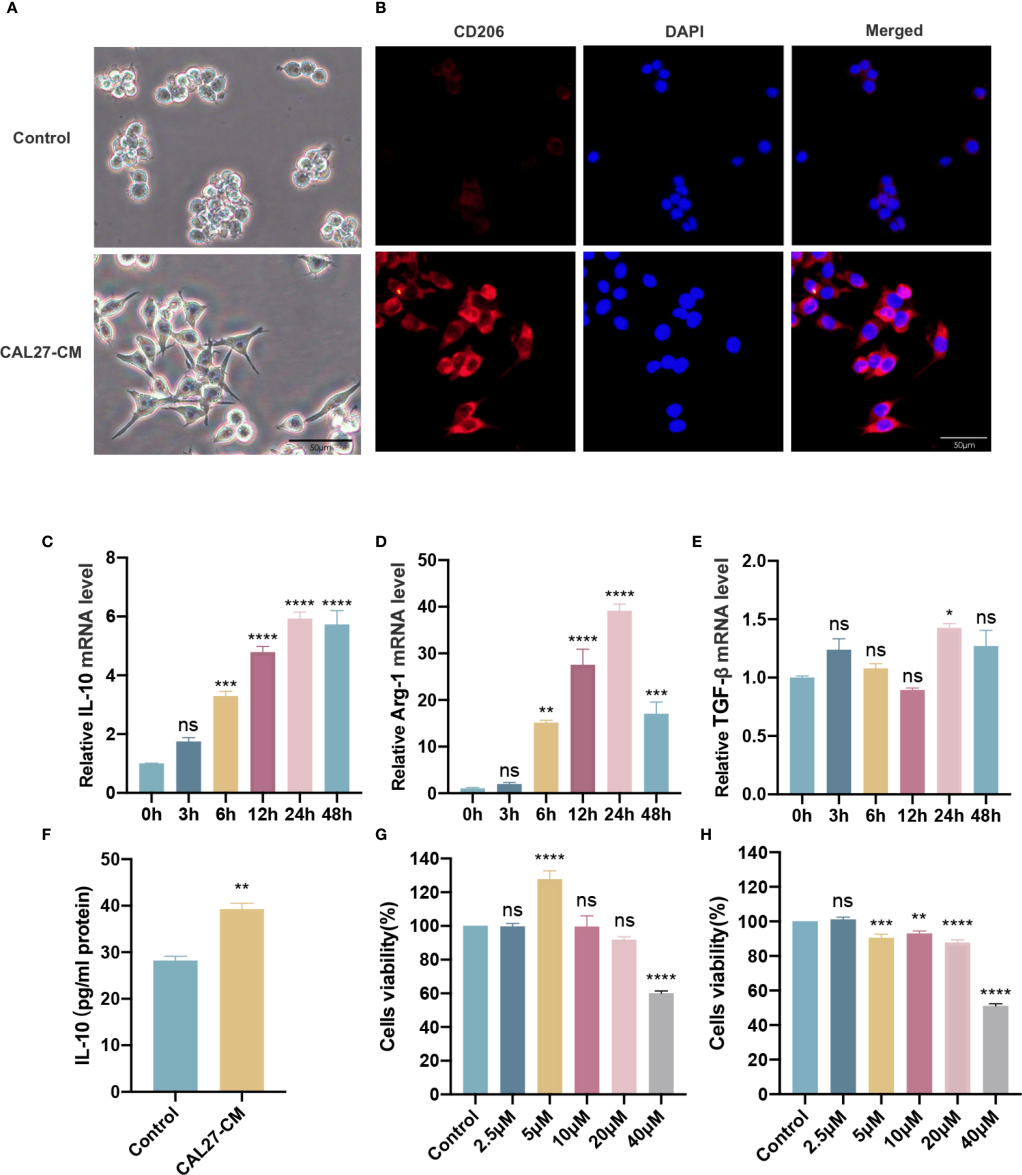
CAL27-CM induced M2 polarization of TAMs. **(A)** Morphological manifestation of TAMs. Macrophages were treated with or without CAL27-CM for 24 h, and then observed under light microscope. **(B)** Immunofluorescence detection of CD206 in TAMs. Macrophages were treated with or without CAL27-CM for 24 h, and then observed under fluorescence microscope. **(C–E)** mRNA expression of IL-10 **(C)**, Arg-1 **(D)**, and TGF-β **(E)** in macrophages treated with CAL27-CM at different time points. **(F)** IL-10 secretion of macrophages treated with CAL27-CM for 24 h. **(G, H)** Cells viability of macrophages and TAMs. Macrophages **(G)** and CAL27-CM-induced TAMs **(H)** were treated with different concentrations of RES (0 μM, 2.5 μM, 5 μM, 10 μM, 20 μM, 40 μM) for 24 h, and the cell viability was detected by CCK-8 assay. Data are presented as the mean ± SD (n = 3). P-values were determined by one-way analysis of variance (ANOVA). (^*^ *P <0.05*; ^**^P <0.01; ^***^P <0.001; ^****^P <0.0001; *ns, P >0.05*). Scale bar: 50 μm.

We used CAL27-CM to induce RAW264.7 cells for 24 h and validate the phenotype of the obtained TAMs. The morphological features of macrophages and TAMs were observed using light microscopy. TAMs were larger, exhibited more pseudopods, and displayed a more dispersed morphology than macrophages, which grew in circular aggregates (**Figure 1A**). We verified the expression of the M2 TAMs surface marker CD206. The results showed that CD206 fluorescence intensity was significantly increased (**Figure 1B**). RT-qPCR results indicated that expression of the M2 marker cytokines IL-10 and Arg-1 mRNA was upregulated in TAMs treated with CAL27-CM in a time-dependent manner, peaking at 24 h and showing a slight decrease at 48 h (**Figures 1C, D**). TGF-β mRNA expression was also significantly upregulated at 24 h compared with other time points (**Figure 1E**). Compared with the control group, IL-10 protein secretion in the CAL27-CM group was significantly increased (**Figure 1F**). These results indicate that 24 h of CAL27-CM treatment induced macrophages into M2-type TAMs.

### 3.2 R-RES and RES inhibited invasion and migration of CAL27 cells through TAMs

First, the optimal concentration of RES was determined by assessing its impact on TAM cell activity. This was achieved using the CCK-8 method, which examined the effects of varying RES concentrations with CAL27-CM (0 μM, 2.5 μM, 5 μM, 10 μM, 20 μM, and 40 μM). The findings showed that the cell survival rate of macrophages (RAW264.7) (**Figure 1G**) and TAMs (CAL27-CM induced RAW264.7 for 24 h) (**Figure 1H**) remained above 80% with RES concentrations up to 20 µM, whereas cell activity was significantly reduced at 40 µM. These results demonstrated that RES concentrations up to 20 µM were safe in this system, and thus the 20 µM was selected for subsequent experiments.

**Figure 2:**
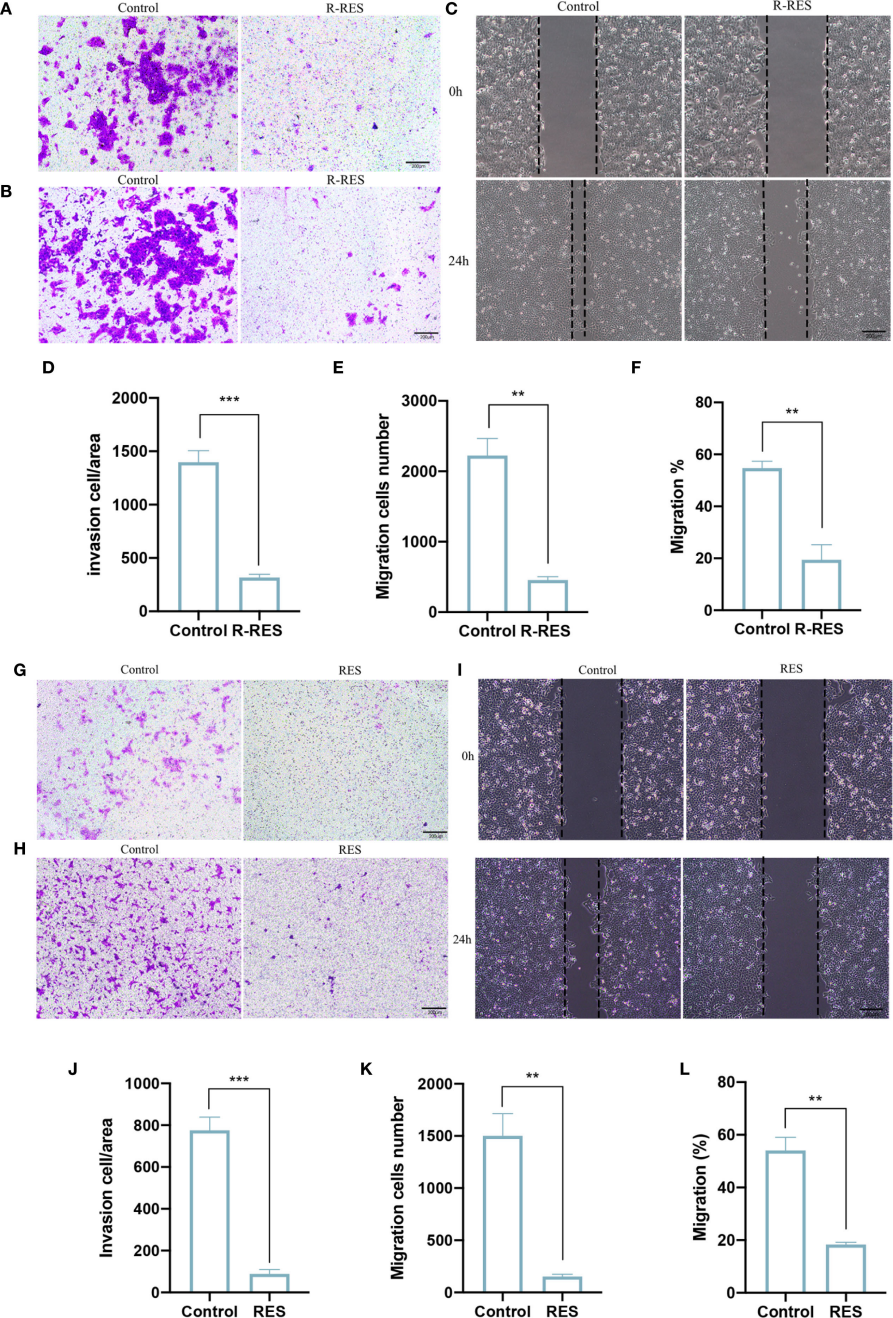
R-RES and RES inhibits invasion and migration of CAL27 cells through TAMs. R-RES group: CAL27-CM was induced for 24 h and then replaced with 20 μM RES **(A–F)**, RES group: 24 h of induction with CAL-CM containing 20 μM RES **(G–L)**. Transwell invasion assay to detect the invasive ability of CAL27 cells **(A, G)** and statistical analysis of the number of invasive cells in each group **(D, J)**. Transwell migration assay was used to detect the migration ability of CAL27 cells **(B, H)** and the statistical analysis of the number of migration cells in each group **(E, K)**. Wound healing assay was used to detect the migration ability of CAL27 cells **(C, I)** and statistical analysis of cell migration rate **(F, L)**. Data are presented as the mean ± SD (n = 3). P-values were determined by t-test. (^**^P <0.01, ^***^P <0.001). Scale bar: 200 μm.

To determine whether RES can influence the invasion and migration of CAL27 cells through TAMs, RES was applied to interfere with TAM polarization in two ways. R-RES refers to RES used for reprogramming TAMs. R-RES group: 20 μM RES was added to TAMs for 24 h. After replacement with complete medium and an additional 24 h of incubation, the TAM supernatant was collected for treatment of CAL27 cells. The Transwell assay results showed that the number of CAL27 cells invading and migrating was substantially decreased in the R-RES group compared with the control group (**Figures 2A, B, D, E**). The wound-healing assay showed that the migration capacity of CAL27 cells in the R-RES group was considerably reduced compared with the control group (**Figures 2C, F**). To investigate whether RES modulates OSCC cell invasion and migration by regulating macrophage polarization, RAW264.7 cells were cultured with 20 μM RES and CAL27 cell culture medium (CAL27-CM) for 24 h. The medium was then substituted with complete medium. After 24 h of incubation, TAM supernatant was collected for treatment of CAL27 cells. Transwell and wound-healing assays showed that the ability of CAL27 cells to invade and migrate was significantly reduced in the RES group compared with the control group (**Figures 2G–L**). The results indicated that RES inhibited the invasion and migration of CAL27 cells by modulating the differentiation of macrophages into TAMs.

### 3.3 R-RES reprogrammed TAMs from M2 to M1 phenotype

**Figure 3:**
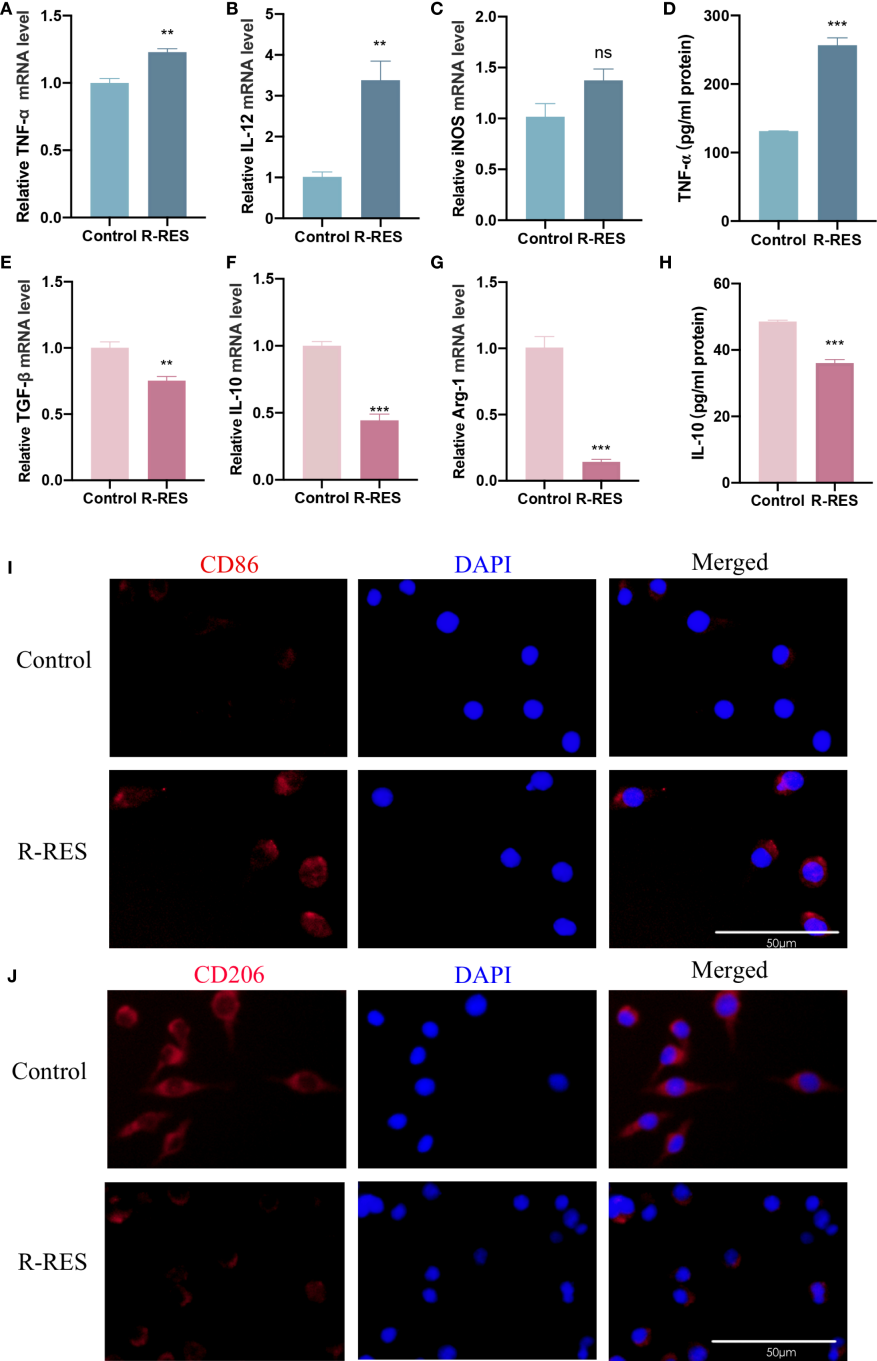
R-RES reprograms TAMs from M2 to M1 phenotype. R-RES group: CAL27-CM was induced for 24 h and then replaced with 20 μM. **(A–E)** Detection of TNF-α **(A)**, IL-12 **(B)**, iNOS **(C)**, TGF-β **(E)**, IL-10 **(F)** and Arg-1 **(G)** mRNA levels in TAMs by RT-qPCR assay **(D, H)** ELISA assay to detect TNF-α **(D)** and IL-10 **(H)** secretion levels in TAMs. **(I, J)** Detection of CD86 **(I)** and CD206 **(J)** expression on the surface of TAMs by immunofluorescence assay. Data are presented as the mean ± SD (n = 3). P-values were determined by t-test. (^**^P <0.01; ^***^P <0.001; *ns, P >0.05).* Scale bar: 50 μm.

To further elucidate the underlying mechanisms, we investigated whether this inhibitory effect of R-RES on the invasion and migration of CAL27 cells was attributable to an altered TAM phenotype. RT-qPCR results indicated that the mRNA levels of the M1 marker cytokines TNF-α and IL-12 were significantly higher, and those of iNOS were slightly higher, in the R-RES group compared with the control group (**Figures 3A–C**). Conversely, the mRNA levels of the M2 marker cytokines TGF-β, IL-10, and Arg-1 were significantly reduced (**Figures 3E–G**). ELISA results showed that the alterations in TNF-α and IL-10 protein secretion levels were consistent with their gene expression levels (**Figures 3D, H**). Immunofluorescence staining showed that the fluorescence intensity of the M2 surface marker CD206 was significantly reduced, while that of the M1 surface marker CD86 was enhanced, in the R-RES group compared to the control group (**Figures 3I, J**). These results indicate that R-RES can reprogram TAMs from the M2 to the M1 phenotype.

### 3.4 RES facilitated M1 polarization of TAMs and suppressed their M2 polarization

**Figure 4:**
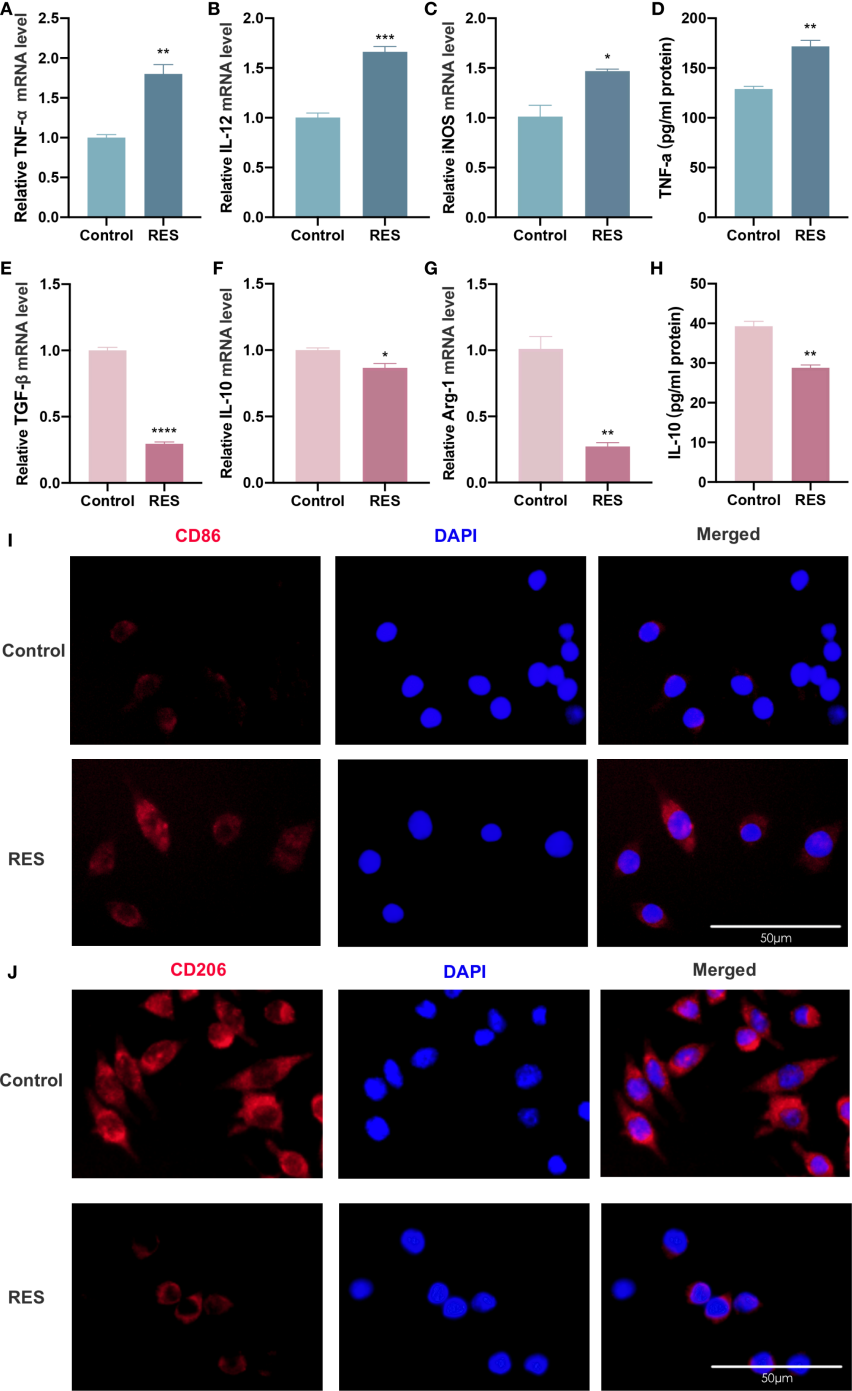
RES promotes M1 polarization of TAMs and inhibits M2 polarization of TAMs. RES group: 24 h of induction with CAL-CM containing 20 μM RES. **(A–F)** Detection of TNF-α **(A)**, IL-12 **(B)**, iNOS **(C)**, TGF-β **(D)**, IL-10 **(E)** and Arg-1 **(F)** mRNA levels in TAMs by RT-qPCR assay **(G, H)** ELISA assay to detect TNF-α **(G)** and IL-10 **(H)** secretion levels in TAMs. **(I, J)** Detection of CD206 **(I)** and CD86 **(J)** expression on the surface of TAMs by immunofluorescence assay. Data are presented as the mean ± SD (n = 3). P-values were determined by t-test. (^*^P <0.05; ^**^ *P <*0.01; ^***^ *P <*0.001; ^****^ *P <*0.0001*).* Scale bar: 50 μm.

The subsequent investigation aimed to ascertain whether RES can also influence the polarization phenotype of TAMs. The polarization phenotype of TAMs was assessed by inducing RAW264.7 cells with CAL27-CM containing RES for 24 h. RT-qPCR results showed that the expression levels of TNF-α, IL-12, and iNOS mRNA in TAMs were considerably higher in the RES group than in the control group (**Figures 4A–C**), while the levels of TGF-β, IL-10, and Arg-1 mRNA were markedly decreased in the RES group compared with the control group (**Figure 4E–G**). ELISA results indicated that the secretion level of TNF-α was significantly higher (**Figure 4D**), while the secretion level of IL-10 was lower (**Figure 4H**) in the RES group compared with the control group. Immunofluorescence staining results showed that surface CD86 expression in TAMs (**Figure 4I**) was enhanced, while CD206 expression (**Figure 4J**) was reduced in the RES group compared with the control group. These results demonstrate that RES promotes M1 polarization of TAMs and suppresses their M2 polarization.

### 3.5 R-RES and RES inhibited activation of Syk signaling pathway in TAMs

**Figure 5:**
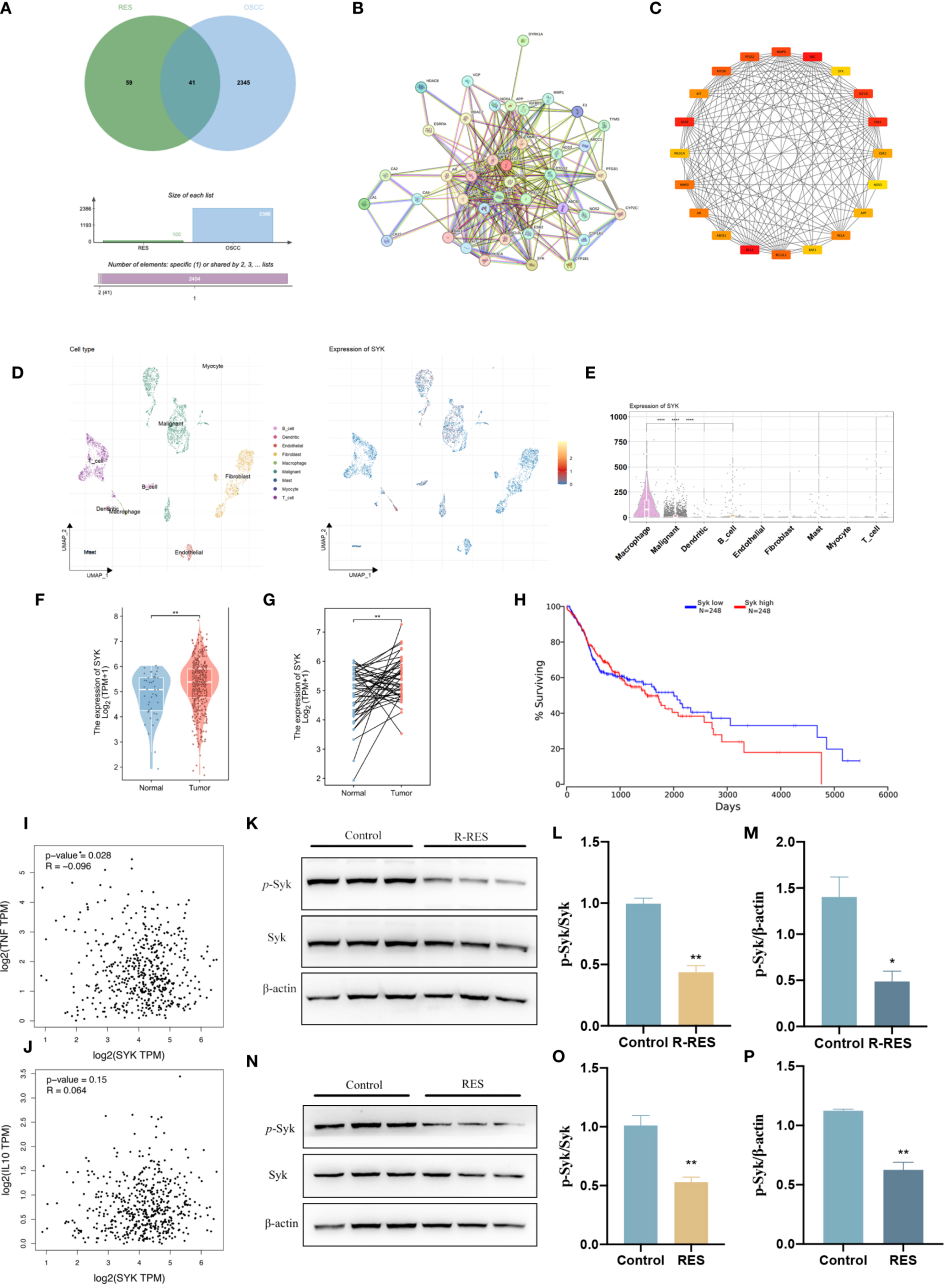
R-RES and RES inhibit Syk phosphorylation in TAMs. R-RES group: CAL27-CM was induced for 24 h and then replaced with 20 μM RES **(A–F)**, RES group: 24 h of induction with CAL-CM containing 20 μM RES. **(A–C)** Wayne plots of targets related to OSCC and RES **(A)**, Protein-protein interaction map of the common targets of OSCC and RES **(B)**, and the top 20 key targets screened by Cytoscape software **(C)**. **(D, E)** UMAP plot showing the cell clusters in the TME and the expression patterns of Syk in different cell types were analyzed using single-cell RNA sequencing data. **(F–G)** The differential expression between tumor and adjacent normal tissues for Syk across all TCGA tumors. **(H)** Correlation between Syk expression levels and overall survival in patients with HNSCC. **(I, J)** In HNSCC tumor, the expression level of Syk correlates with TNF (TNF-α) **(I)** and IL-10 **(J)** were analyzed by GEPIA (*Pearson* correlation was selected). **(K–P)** Statistical analysis of protein expression and relative protein expression of Syk and p-Syk in R-RES **(K–M)** and RES **(N–P)** group TAMs. Data are presented as the mean ± SD (n = 3). P-values were determined by t-test. (*P <0.05; **P <0.01).

The mechanisms by which RES regulates TAM polarization were further investigated. A total of 2,345 OSCC-related targets were identified using the GeneCards database, 59 RES-related target genes were obtained from the SwissTargetPrediction platform, and a “RES-OSCC” intersection target map was generated with the Bioinformatics & Evolutionary Genomics network platform, yielding 41 target genes (**Figure 5A**). The STRING V11.0 was used to analyze the proteins among the 41 intersection targets and to construct the protein–protein interaction network (**Figure 5B**). In the figure, nodes represent proteins, and connecting lines illustrate their relationships. The top 20 key target proteins were identified based on degree value, including spleen tyrosine kinase (Syk) (**Figure 5C**). To investigate the site of Syk expression in HNSCC tissues, a single-cell dataset (38; https://www.weizmann.ac.il/sites/3CA/) was used for further analysis (38). UMAP analysis was conducted to reduce the dimensionality and cluster the cells into nine cell populations (**Figure 5D**). Syk expression was highest in macrophages (**Figure 5E**). In addition, analysis of The Cancer Genome Atlas (TCGA) database showed that Syk is highly expressed in HNSCC tumor tissues compared with normal tissues (**Figures 5F, G**). Using the TCGA database, we examined the correlation between Syk expression and overall survival in HNSCC patients (**Figure 5H**). In the correlation analysis, Syk showed a weak positive correlation with IL-10 (M2 TAM marker; *R* = 0.064, *P* < 0.05) and TNF-α (M1 TAM marker; *R* = −0.096, *P >*0.05) (**Figures 5I, J**). We examined Syk protein levels and phosphorylation in TAMs after RES intervention by Western blot assay. The findings revealed no significant changes in total Syk protein levels and significantly lower p-Syk protein expression in the R-RES group compared with the control group (**Figures 5K–M**), as well as no significant changes in total Syk protein levels and significantly lower p-Syk expression in the RES group compared with the control group (**Figures 5N–P**). These results indicate that R-RES and RES inhibit Syk phosphorylation.

### 3.6 Syk inhibitor facilitated M1 polarization of TAMs and suppressed their M2 polarization

**Figure 6:**
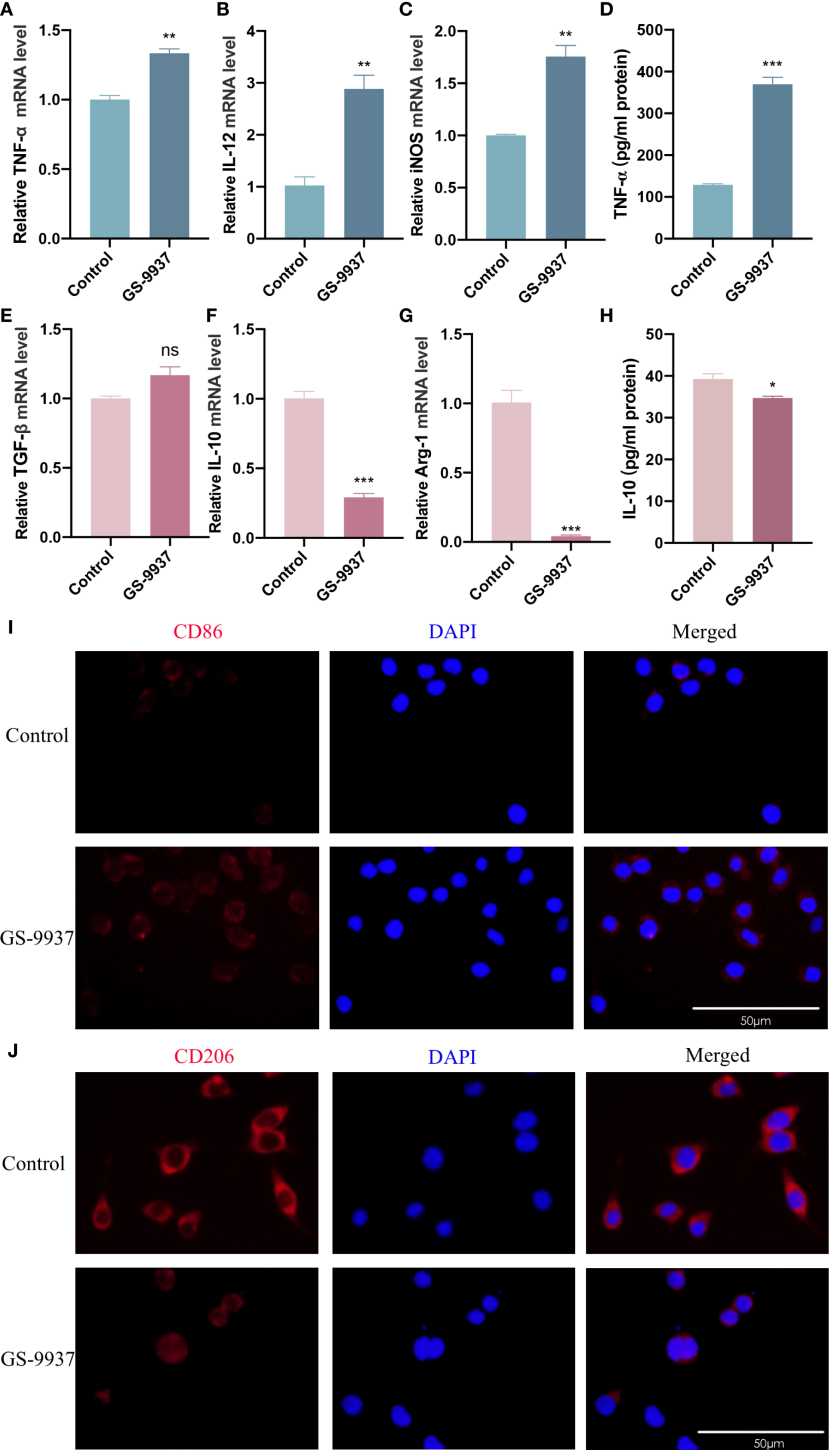
Syk inhibitor promotes M1 polarization of TAMs and inhibits M2 polarization of TAMs. GS-9937 (1 μM) was used to inhibit the activation of Syk pathway in TAMs. (**A–C, E–G**) RT-qPCR assay to detect TNF-α **(A)**, IL-12 **(B)**, iNOS **(C)**, TGF-β **(E)**, IL-10 **(F)** and Arg-1 **(G)** mRNA levels in TAMs. **(D, H)** ELISA assay to detect TNF-α **(D)** and IL-10 **(H)** secretion levels in TAMs. **(I, J)** Detection of CD86 **(I)** and CD206 **(J)** expression on the surface of TAMs by immunofluorescence assay. Data are presented as the mean ± SD (n = 3). P-values were determined by t-test. (*P <0.05; **P <0.01; ***P <0.001; ns, *P >*0.05). Scale bar: 50 μm.

GS-9937 is a selective Syk inhibitor currently under evaluation in phase II clinical trials for hematological malignancies. It has demonstrated a favorable *in vitro* and *in vivo* selectivity profile with fewer dose-limiting adverse effects (39). RAW264.7 cells were treated with CAL27-CM containing the Syk inhibitor GS-9937 for 24 h to assess the polarization phenotype of TAMs. RT-qPCR showed that the expression levels of TNF-α, IL-12, and iNOS mRNA in TAMs were significantly increased in the GS-9937 group compared with the control group (**Figures 6A–C**). There was no significant difference in TGF-β mRNA expression between the two groups (**Figure 6E**). IL-10 and Arg-1 mRNA levels were significantly lower in the GS-9937 group compared with the control group (**Figures 6F, G**). ELISA results showed that TNF-α secretion level was significantly enhanced (**Figure 6D**), while IL-10 secretion was decreased (**Figure 6H**) in the GS-9937 group compared with the control group. Immunofluorescence staining indicated that surface CD206 expression in TAMs was diminished, whereas CD86 expression was enhanced in the GS-9937 group compared with the control group (**Figures 6I, J**). These results indicate that inhibition of the Syk signaling pathway in TAMs promoted M1 polarization and suppressed M2 polarization.

### 3.7 Syk inhibitor attenuated invasive and migratory ability of CAL27 cells

**Figure 7:**
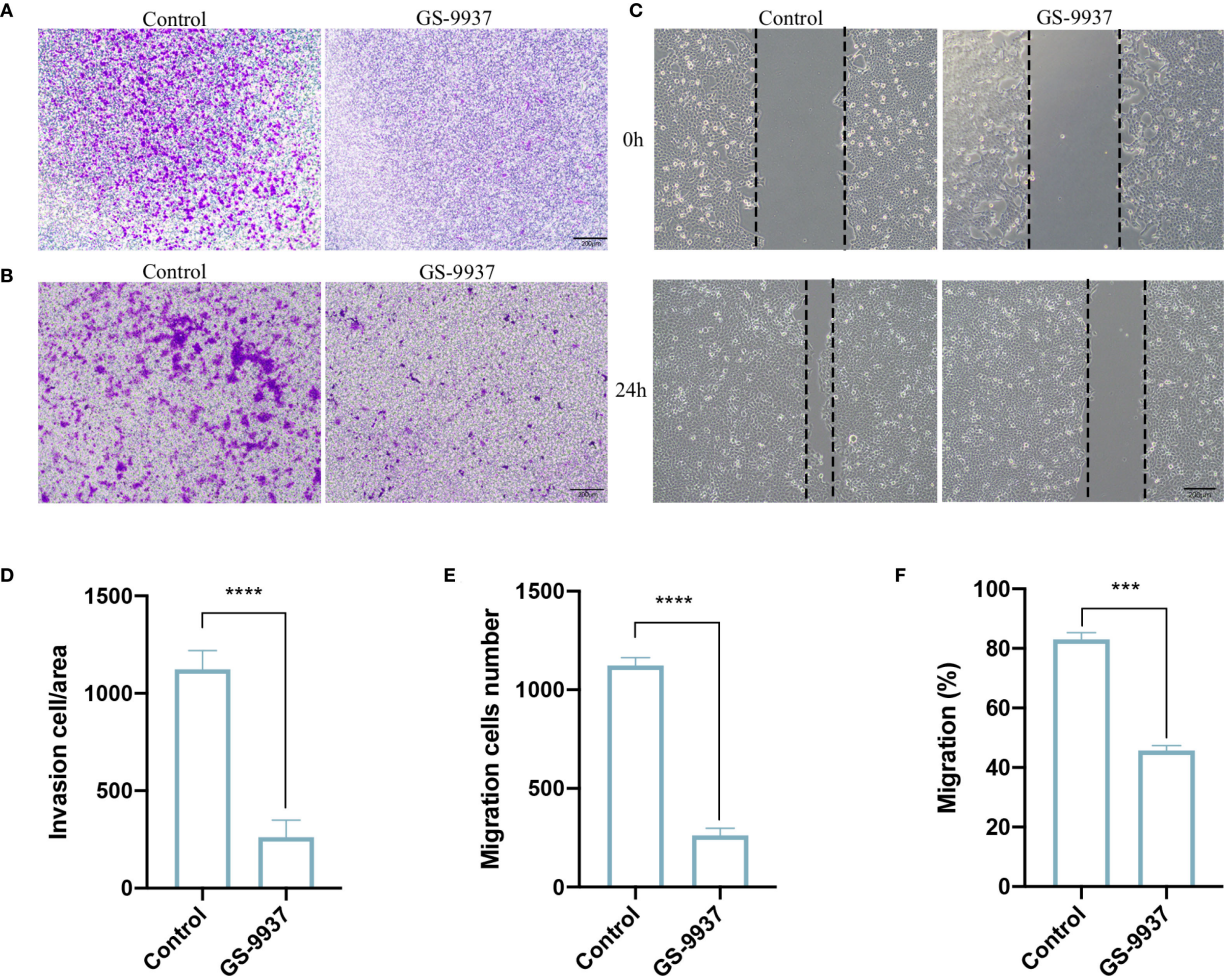
Syk inhibitor attenuates invasive and migratory ability of CAL27 cells. **(A, D)** Transwell assay to detect the number of invasive cells in each group of CAL27 cells and statistical analysis. **(B, E)** Transwell assay to detect the number of migrating cells in each group of CAL27 cells and statistical analysis. **(C, F)** Cell scratch assay to detect the migration ability of CAL27 cells in each group and statistical analysis. Data are presented as the mean ± SD (n = 3). P-values were determined by t-test. (***P <0.001; ****P <0.001). Scale bar: 200 μm.

We next verified whether the inhibition of the Syk signaling pathway in TAMs attenuated the invasion and migration of CAL27 cells. TAMs were treated with GS-9937. The results of Transwell assay revealed a significant reduction in the number of CAL27 cells that invaded and migrated in the GS-9937 group compared with the control group (**Figures 7A, B, D, E**). The wound-healing assay demonstrated that the migration ability of CAL27 cells in the GS-9937 group was markedly reduced compared with the control group (**Figures 7C, F**). These results indicate that the invasion and migration ability of CAL27 cells can be diminished by inhibiting the Syk signaling pathway in TAMs.

## 4 Discussion

Plant extracts can exert anti-tumor effects by regulating the polarization phenotype of TAMs (18–20). In this study, we found that CAL27-CM induced M2 polarization of TAMs. R-RES and RES inhibited the invasion and migration of CAL27 cells through TAMs. R-RES reprogrammed TAMs from the M2 to the M1 phenotype. RES facilitated M1 polarization of TAMs and suppressed their M2 polarization. Mechanistically, R-RES and RES inhibited the activation of the Syk signaling pathway in TAMs. Inhibition of the Syk signaling pathway in TAMs attenuated the invasive and migratory ability of CAL27 cells by facilitating M1 polarization and suppressing M2 polarization.

The polarization of TAMs can be induced by the CM of OSCC cells or by a co-culture system (18, 40). The co-culture system is favored because OSCC cells continuously secrete mediators that induce the polarization of TAMs (20, 41). However, the influence of drug-regulated OSCC cells on TAMs is inevitable when continuous drug intervention targeting TAMs is applied during the co-culture period. In contrast, TAM polarization induced by OSCC cell CM can effectively address this drawback. Therefore, CAL27-CM was used to induce TAM polarization in this study. We found that CAL27-CM induced M2 polarization of TAMs, further confirming previous studies (12, 42).

RES has been shown to suppress OSCC invasion and migration through different mechanisms (27–29). However, studies on RES intervention in OSCC invasion and migration have focused on its direct effects on OSCC cells, with little attention to its indirect effect through TAMs. In this study, RES modulated the polarization of TAMs in two ways to examine whether it could influence OSCC invasion and migration through TAMs. R-RES was added after 24 h induction of macrophages with the CAL27-CM. RES and CAL27-CM were added simultaneously to observe changes in polarization phenotype of TAMs. Our results showed that R-RES and RES inhibited the invasion and migration of CAL27 cells by regulating TAM polarization.

RES could reprogram M2 TAMs to the M1 phenotype in a tumor model of lung adenocarcinoma (4). RES reversed macrophage polarization and increased the M1/M2 polarization ratio in breast cancer (32). RES can convert macrophages to the M1 phenotype in the lungs of TNBC-bearing mice (43). The ethanol extract of peanut sprout tea containing RES suppressed the interaction between breast cancer cells and TAMs, promoting M1 TAMs while inhibiting M2 TAMs (44). However, the effects of RES on TAM polarization in OSCC have been rarely reported. In this study, RES regulated TAM polarization through a dual pathway. R-RES reprogrammed TAMs from the M2 to the M1 phenotype. RES facilitated M1 polarization of TAMs while suppressing their M2 polarization. These results are consistent with previous studies, further highlighting the impact of RES on tumor immunology.

Inflammation-related studies have revealed the signaling pathways through which RES regulates macrophage polarization (45). RES might promote M2 polarization of macrophages after myocardial infarction via the JAK2/STAT3 signaling pathway (46, 47). RES can regulate macrophage polarization via the TLR4/MyD88 signaling pathway (48). Polydatin, a glucoside of resveratrol, remodels macrophage polarization via the NF-κB signaling pathway (49). A resveratrol-mediated hydrogel can modulate macrophage polarization via the PI3K/AKT signaling pathway (50, 51). Few reports have evaluated the signaling pathway in TAMs regarding the mechanism by which RES regulates TAM polarization.

Syk is a non-receptor tyrosine kinase involved in cancer progression (52, 53). RES has been reported to inhibit Syk phosphorylation in monocytes/macrophages during inflammation. RES suppresses monosodium urate-induced inflammation by inhibiting Syk phosphorylation in monocytes (54). Amurensin H, a RES dimer, alleviates LPS-induced inflammation by inhibiting the Syk/NF-κB pathway in macrophages (55). However, the regulation of Syk activation by RES has not been reported in TAMs. In this study, bioinformatics analysis showed that Syk was among the top 20 key target proteins of OSCC and RES. UMAP and TCGA database analyses further revealed that Syk was predominantly expressed in macrophages of HNSCC. These results suggested that Syk may be involved in the regulation of TAM polarization by RES. We then verified whether RES regulated the Syk signaling pathway in TAMs. The results showed that R-RES and RES suppressed the activation of Syk signaling pathway in TAMs. Intervention experiments with Syk inhibitors further demonstrated that inhibiting the Syk signaling pathway in TAMs attenuated the invasive and migratory abilities of CAL27 cells by facilitating M1 polarization of TAMs and suppressing their M2 polarization. Targeting Syk in TAMs may represent a promising treatment strategy for cancers.

Subsequent experiments will focus on identifying the upstream receptors and downstream signaling pathways through which RES exerts its effects on TAMs. Toll-like receptors (TLRs) are pathogen recognition receptor that trigger intracellular signaling cascades in response to pathogens, leading to the secretion of interferons and proinflammatory cytokines and the activation of host defense programs necessary for innate or adaptive immune responses (56). TLR4 is one of the most widely studied TLRs in the tumor microenvironment and plays a key role in immune surveillance and tumor progression (57, 58). Our previous results showed that ENO1 and HSP27 regulate TAM polarization and cytokine secretion through the TLR4 on the surface of TAMs (7, 12). Several studies have reported that RES regulates the expression of TLR4 and Syk is recruited to the TLR4-related receptor complex (59, 60). RES suppressed MMP3 and MMP9 expression and secretion by inhibiting the TLR4/Syk/NLRP3 inflammasome pathway in platelets (61). STRING database analysis confirmed the association of TLR4 and Syk (**Supplementary Figure S1A**). TCGA database analysis showed a positive association between TLR4 and Syk (**Supplementary Figure S1B**). In this study, RES may regulate TAM repolarization through TLR4.

Amurensin H exerts anti-inflammatory and chondroprotective effects *in vivo* and *in vitro* and inhibits TLR4/Syk/NF-κB signaling in chondrocytes (62). In human proximal tubular epithelial cells, high glucose triggers the immediate, ROS-dependent release of HMGB-1 into the extracellular space, thereby activating the TLR4/MyD88/Syk/NF-κB pathway (63). Key molecules in TLR4 downstream signaling in mice with retinal ischemia/reperfusion injury are Syk and NF-κB (64). TREM1 enhances microglial plasticity through the Syk/PDK/STAT3 signaling axis, thereby promoting an immune environment favorable to tumor progression (65). RES suppressed MMP3 and MMP9 expression and secretion by inhibiting the TLR4/Syk/NLRP3 inflammasome pathway in platelets (61). STRING database analysis confirmed the association of Syk/NF-κB, Syk/STAT3, and Syk/NLRP3 (**Supplementary Figure S1A**). The results of TCGA database showed the positive association of Syk/NF-κB and Syk/STAT3 (**Supplementary Figures S1C–E**). We speculate that RES may regulate TAM polarization through TLR4/Syk/NF-κB or TLR4/Syk/STAT3 signaling pathways in OSCC (66).

Although RES targets multiple pathways, its rapid metabolism and low oral bioavailability restrict its clinical application. To overcome these disadvantages, RES has been encapsulated in various nanocarriers, including liposomes, polymeric nanoparticles, solid lipid nanoparticles (SLNs), protein-based nanoparticles, and inorganic nanoparticles. These can modulate the release of the drug to achieve the desired effect. Significant therapeutic concentrations have been demonstrated in plasma, with improved bioavailability (67). Polymer nanoparticles are the most widely used among these nanocarriers due to their high encapsulation efficiency. This significantly reduces the number of nanocarriers required to achieve the desired bioactivity, while also reducing the risk of toxicity and side effects (68). Literature indicates that Ancic et al. conducted a study investigating the use of resveratrol nanoparticles as an anti-tumor agent in mice with Ehrlich ascites tumor (EAT) (69). It is necessary to incorporate preliminary pharmacokinetic and toxicity evaluations, and to explore nano-formulation and targeted delivery strategies for improving translational prospects.

Moreover, during the early tumorigenesis process, M2 TAMs can significantly promote the tumor survival, growth, and metastasis by causing immunosuppression of CD8+ T cells and creating a tumor-favorable microenvironment (70). M2 TAMs, together with regulatory T cells (T-regs), are reprogrammed to become immunosuppressive. This results in the inactivation or impaired recruitment of cytotoxic CD8 + T and Natural Killer (NK) cells (71). M2-like macrophages drive tumor growth both directly and indirectly by suppressing cytotoxic cell populations, including CD8+ T cells and NK cells (72). Previous research indicated that RES improved CD8+ T cell cytotoxicity by increasing TNF-α, IFN-γ, IL-12, and IL-2 (73). Consequently, we hypothesize that the reduction of RES-induced M2-phenotype TAMs may be associated with CD8+ T cells. This hypothesis will be tested in future experimental research.

The anti-tumor and pro-tumor phenotypes of TAMs make them a double-edged sword in OSCC progression, while their phenotypic plasticity also makes them important potential therapeutic targets. We found that RES suppressed OSCC invasion and migration through CAL27-CM-induced TAMs. Mechanistically, RES reprogrammed TAMs from a pro-tumor to an anti-tumor phenotype and promoted macrophage polarization toward the anti-tumor phenotype by inhibiting the activation of the Syk signaling pathway. In conclusion, RES suppresses OSCC invasion and migration by regulating TAM polarization through the Syk signaling pathway, further elucidating its anti-tumor mechanism. Targeting Syk in TAMs may represent a promising therapeutic strategy for cancer. As a preliminary mechanistic study, the present study provides directions for future research. The regulatory effects of RES on TAM polarization and OSCC invasion and migration, along with the related upstream receptors and downstream signaling pathways, need to be further verified in human monocyte-derived macrophages (MDMs), primary TAMs isolated from OSCC patients, and murine xenograft models or spontaneous OSCC models. Nanoformulated RES (74, 75) and RES-loaded nanocarriers (76–78) targeting TAMs may represent effective treatment strategies against OSCC progression.

## Data Availability

The original contributions presented in the study are included in the article/**Supplementary Material**, further inquiries can be directed to the corresponding authors.
