# Supplementary material for: Resveratrol suppresses OSCC invasion and migration by regulating macrophage polarization via Syk signaling pathway
Source: Front Immunol. 2025 Sep 29;16:1660851. doi: 10.3389/fimmu.2025.1660851 (PMC12515654; doi:10.3389/fimmu.2025.1660851)
Supplement: Supplementary file 1 [file SupplementaryFile1.docx]

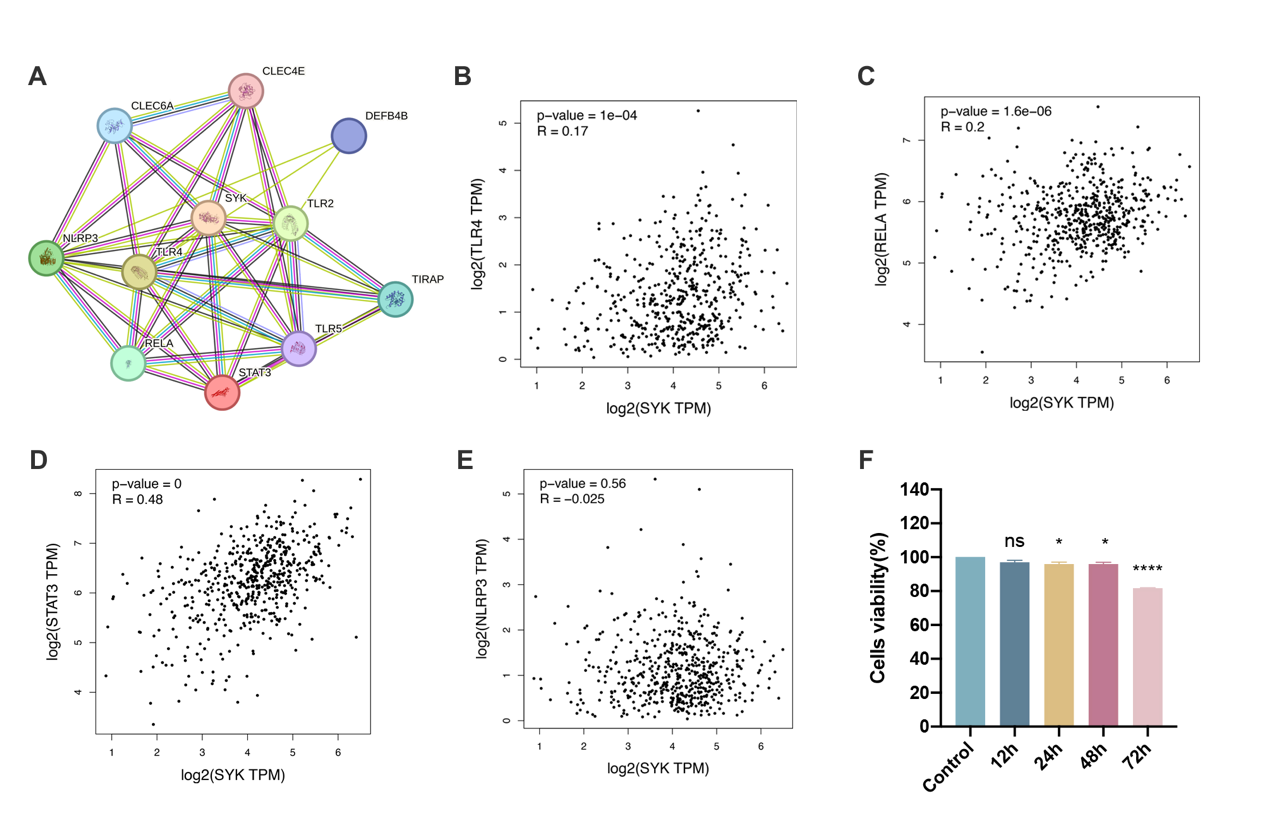


**Figure S1. RES may regulate TAMs polarization through TLR4/Syk/NF-κB signaling pathway.** (A) The STRING database is used for the construction of protein-protein interactions (PPIs) for proteins interacting with Syk. (B- E) The Gene correlations between Syk and TLR4, NF-κB (RELA), NLRP3, and STAT3 in the GEPIA database. (F) Cells viability of RAW264.7 after being induced with CAL27-CM for 0, 12, 24, 48, 72 h, and the cell viability was detected by CCK-8 assay. Data are presented as the mean ± SD (n=3). P values were determined by one-way analysis of variance (ANOVA). (*P < 0.05; ****P < 0.0001; ns, P>0.05).
